# Complete avulsion of the heel pad with talar and calcaneal fracture: salvage with multiple K-wire anchorage, internal fixation and free ALT flap

**DOI:** 10.1007/s00402-022-04439-9

**Published:** 2022-04-25

**Authors:** J. Herold, K. Kamin, O. Bota, A. Dragu, S. Rammelt

**Affiliations:** University Center of Orthopaedic, Trauma and Plastic Surgery, University Hospital Carl Gustav Carus, Dresden, Germany

**Keywords:** Degloving, Planta, Sole, K-wires, Free flap, Reconstruction, Hindfoot

## Abstract

**Background:**

Degloving of the sole of the foot is a rare and serious injury because the heel pad cannot be replaced by similar tissue. The management is challenging and only a few cases have been reported with different treatment regimens.

**Methods:**

Here, we report on a 46-year-old female patient with complex foot trauma consisting of complete avulsion of the heel pad at the hindfoot and a soft tissue defect at the posterior aspect of the heel accompanied by rupture of the anterior tibial tendon and fractures of the talus, calcaneus and midfoot. The sole of the foot was fixed to the calcaneus with multiple temporary Kirschner wires and moist wound dressings. The anterior tibial tendon was sutured. The soft tissue defect at the posterior heel was treated with a free anterolateral thigh flap. The fractures were fixed in staged procedures.

**Results:**

At 2-year follow-up, the patient had a durable soft tissue cover over the heel with full sensation over the sole and a pliable flap over the posterior aspect of the heel. The patient was able to fully bear weight and was pain free during her daily activities in comfortable, custom shoes. All fractures had healed, the talar neck fracture after one revision and bone grafting. The foot was plantigrade and stable with preserved painless but limited range of motion at the ankle, subtalar and mid-tarsal joints.

**Conclusion:**

The unique tissue at the sole of the foot can be salvaged even in cases of full degloving at the hindfoot with the simple method of anchorage with multiple temporary K-wires. Traumatic defects of the vulnerable skin at the posterior aspect of the heel requires durable coverage with free flap coverage. With staged treatment of all bone and soft tissue injuries, a favorable result can be obtained even in case of a complex foot trauma.

## Introduction

Degloving injuries of the sole represent an uncommon, but severe injury of the foot with a poor prognosis [1–3]. Partial or complete avulsion injuries of the heel pad are most commonly caused by a crush injury like road traffic accidents and therefore are often associated with other skeletal and soft tissue injuries [4, 5]. The typical mechanism of trauma includes a separation of the flap in a posterior to anterior direction by blunt tangential forces, leaving only an anterior soft tissue bridge [4]. As a result, the soft tissues are disrupted from the serving of blood supply, leaving vital structures like nerves, vessels, tendons and bone exposed [6]. Due to the complex anatomy of the heel pad, the treatment and the reconstruction of this highly specialized tissue imposes a great challenge on the treating surgeon.

The heel pad (planta pedis) represents a unique anatomical structure. During walking and running, the soft tissues of the sole are exposed to extreme shearing stresses and axial loading while permitting sensibility and durability [7]. This can be achieved by a “water cushion” or “honeycomb” architecture of the subcutaneous tissue. The plantar fat is interwoven by a complex structured system of collagen and elastic fibers, which permits pressure distribution within the tissues. The epidermis and the cornification of the sole is thicker than in every other region of the body [8].

Many investigators have declared the degloved skin as initially dead, thus advocating debridement and covering the underlying tissue with skin grafts or flaps [5, 9–14]. However, considering the aim of tissue reconstruction, following the rule to replace like with like tissues any reconstruction of the plantar sole with alternative tissue is less than optimal [15]. Due to the lack of durability of the reconstructed tissue and the failure to provide a stable weight-bearing area, skin grafts, fasciocutaneous or muscle flaps are prone to soft tissue complications like atrophy, hyperkeratosis, instability, ulceration, resulting in chronic heel pain and a disturbed gait [7, 12].

In an uncommon but severe injury the choice of operative treatment is not only based on the extent of the injury, but also on the experience and the preferences for a particular method of the treating surgeon [6]. In cases of complete or subtotal avulsions of the heel pad with extensive neurovascular damage, the sole will need replantation with the use of microvascular surgical techniques [4, 16]. If the vascular supply remains intact, reattachment of the avulsed heel pad may be an option. However, data about heel-preserving strategies are scarce.

To our knowledge, there has been two reported salvage strategies with promising results for a subset of these injuries. Cantrell et al. and Ozturk et al. reported of strategies consisting of defatting the sole, adding fenestration for wound drainage and treating it as a skin graft with negative pressure wound therapy. Cantrell et al. additionally advocated fixing the graft with K-wires [17, 13].

For partial avulsions with an intact vascularization and sensation, different strategies have been described for heel pad reattachment. After simple suturing of the sole complications like wound breakdown, infection and flap necrosis have been reported [18–20]. Anecdotally, promising results have been achieved with anchorage of the heel pad with the use of multiple K-wires or anchor fixation in partial avulsions [1, 12, 14].

Here, we report successful salvage of a complete degloving injury at the heel with reattachment of the heel pad with sutures and temporary K-wire anchorage supplemented by a free flap to cover the necrotic skin over the Achilles tendon insertion and staged fixation of a talar and calcaneal fracture in a complex foot trauma. To our knowledge, this is the first report of a combination of K-wire anchoring and microsurgical reconstruction using a free flap. The patient has been informed that her case would be submitted for publication and she provided consent.

## Case report

**Fig. 1:**
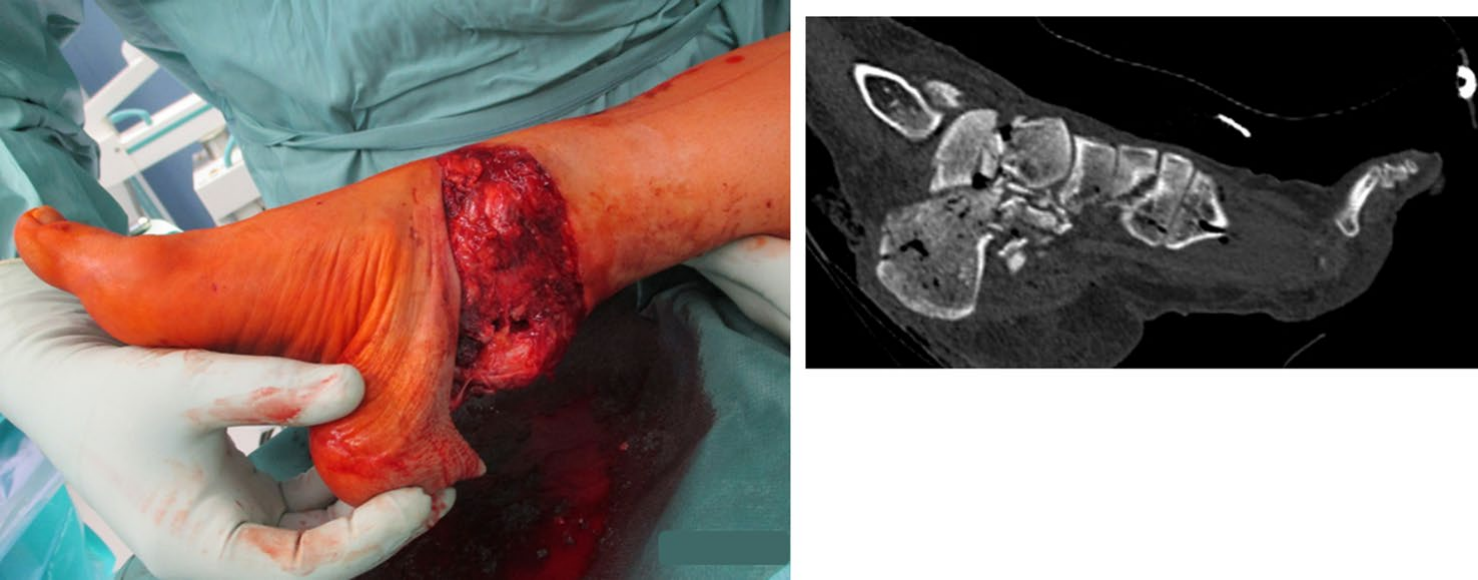
**a** Clinical findings of the complete degloving of the heel pad. **b** The initial CT scan obtained in the emergency department revealed a displaced intra-articular fracture of the calcaneus, displaced fractures of the talar neck and lateral talar process, non-displaced fractures of the navicular, first cuneiform and first metatarsal base and a subluxation at the mid-tarsal joint

A 46-year-old woman was presented to our trauma center after being involved in a motorcycle accident. Initial assessment at the emergency department revealed a complex trauma to her right foot and ankle including a displaced intra-articular fracture of the calcaneus (sustentacular fracture and comminuted anterior process fracture), displaced fractures of the talar neck and lateral talar process, non-displaced fractures of the navicular, first cuneiform and first metatarsal base and a subluxation at the mid-tarsal (Chopart) joint. There was a complete degloving of the heel pad and the soft tissues around the medial and lateral aspect of the hindfoot leaving the calcaneus, both malleoli and the Achilles tendon insertion uncovered (Fig. 1a, b).

**Fig. 2:**
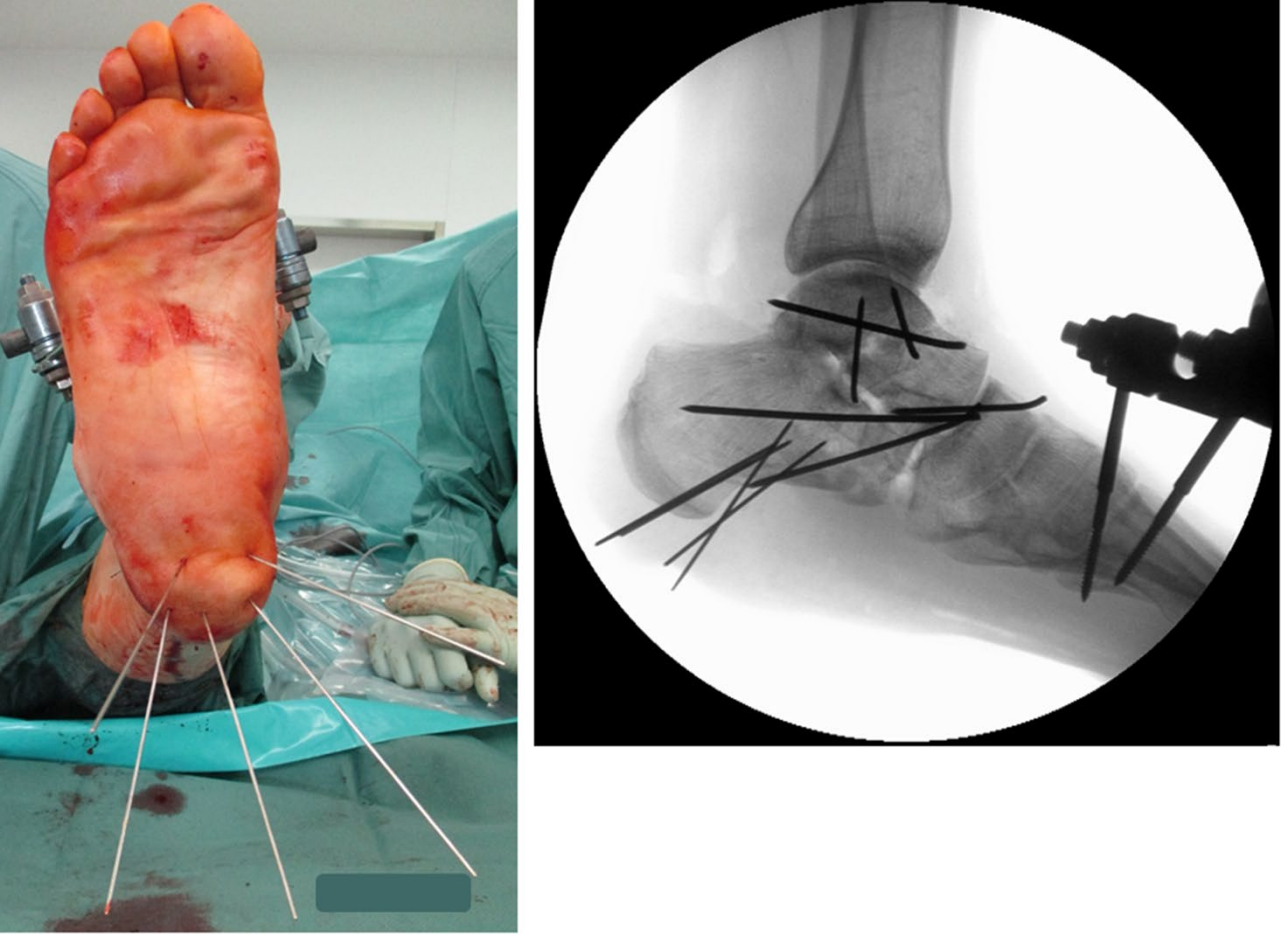
**a** Anchoring of the avulsed heel pad to the tuberosity of the calcaneus using multiple K-wires. **b** Fracture reduction was achieved via a small lateral approach over the sinus tarsi under direct vision using K-wires. The unstable talonavicular joint was reduced and transfixed with a K-wire

The patient was immediately taken to the operating room for thorough debridement and lavage of the wound. Via a small lateral approach over the sinus tarsi the lateral process fracture, the anterior process fracture of the calcaneus and the talar neck fracture were reduced under direct vision on the subtalar joint and fixed with K-wires. The unstable talonavicular joint was reduced and transfixed with a K-wire. Upon exploration of the medial wound, a complete rupture of the anterior tibial tendon was detected and treated with a direct suture. The avulsed heel pad was anchored to the tuberosity of the calcaneus using multiple K-wires. The distance between the wires was set at 2 cm to ensure a stable fixation without compromising the blood supply to the heel pad. The wires were placed at a safe distance to the skin wound edge in order not to interfere with wound healing (Fig. 2a, b). The wires were cut short to facilitate the application of sterile dressing. The wound edges were adapted without tension at the margins and a collagen-based skin substitute (Epigard, Biovision GmbH, Ilmenau Germany) was applied over the remaining skin defect over the Achilles tendon insertion. A tibiometatarsal external fixator was applied to facilitate soft tissue consolidation.

**Fig. 3:**
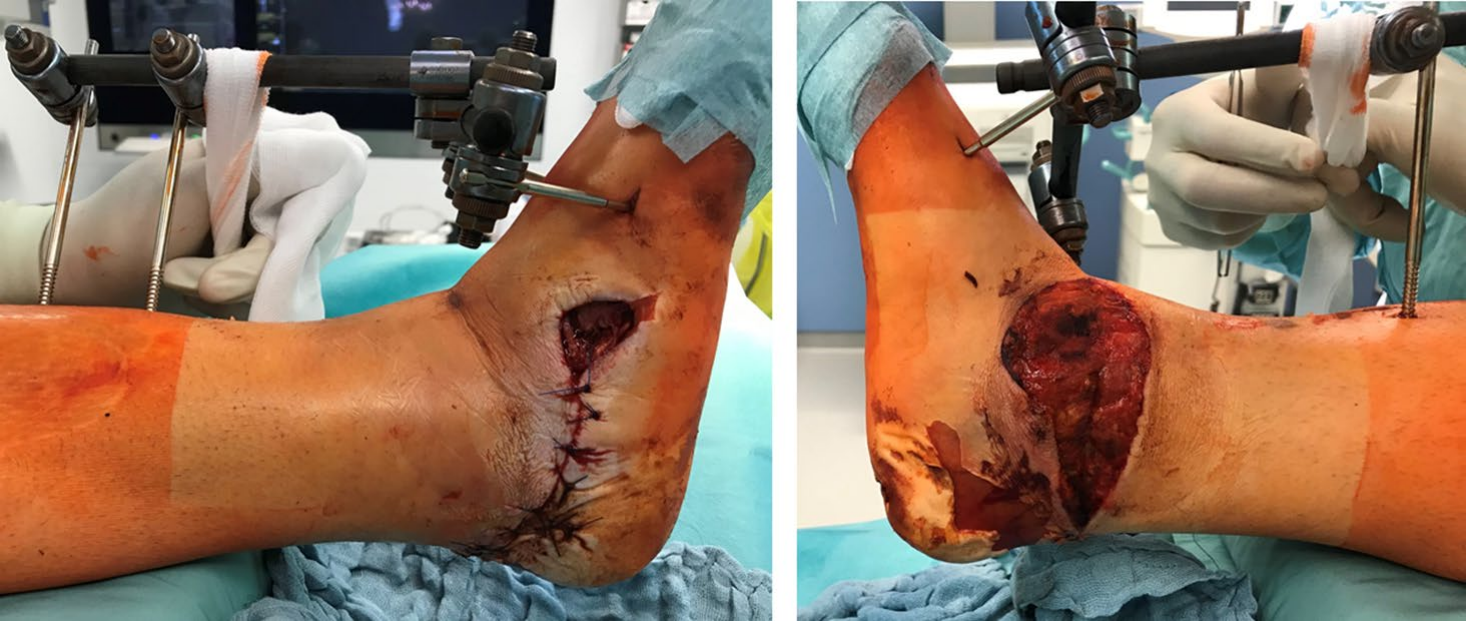
**a** Repeated debridements were carried out as well as the application of a negative pressure wound therapy to promote healing. **b** After complete resection of all necrotic tissue, a full thickness soft tissue defect above the Achilles tendon insertion and malleoli of 26 × 7 cm resulted

An intravenous antibiotic was administered and dressings changed regularly. The patient was returned to the operating room for repeated debridements of the wound edges over the Achilles tendon and malleoli, followed by the application of a negative pressure wound therapy to promote healing. After complete resection of all necrotic tissue, a full thickness soft tissue defect above the Achilles tendon insertion and malleoli of 26 × 7 cm resulted, necessitating free flap coverage (Fig. 3a, b).

**Fig. 4:**
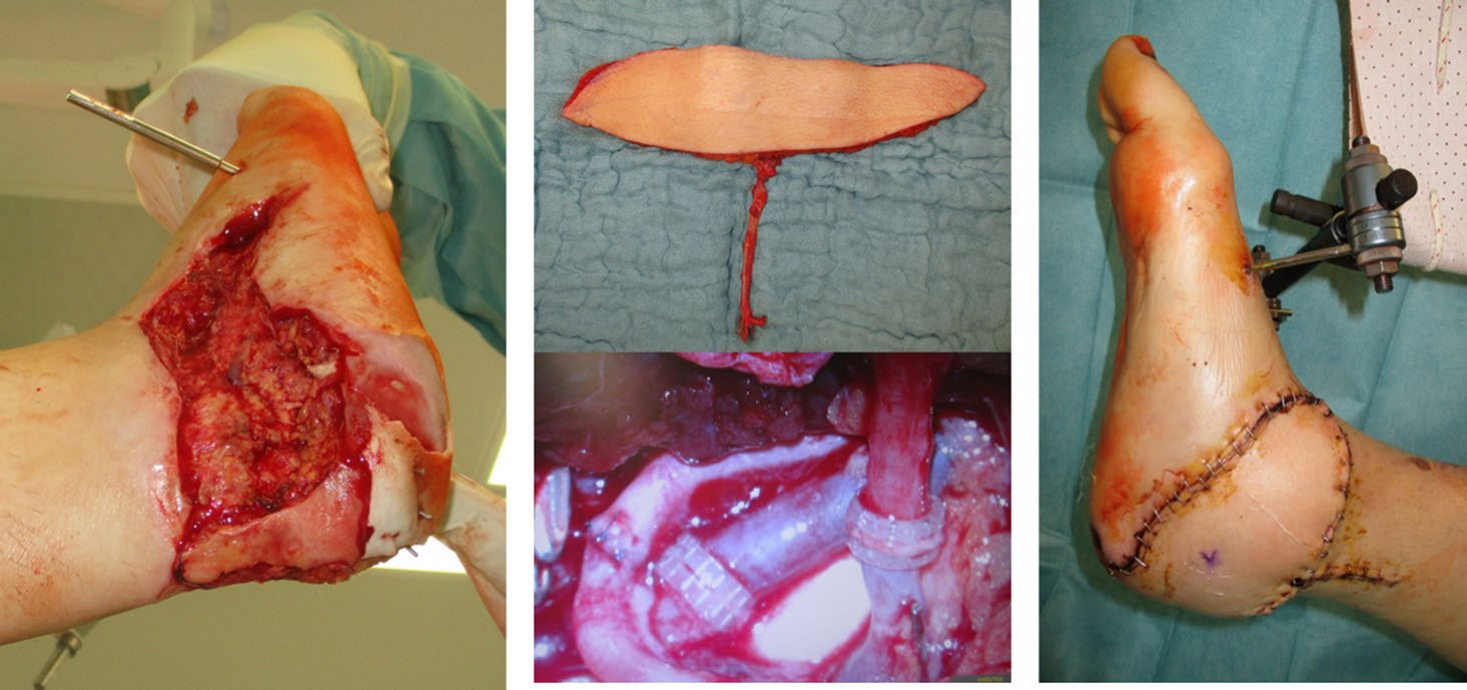
**a** The remaining soft tissue defect necessitating flap coverage. **b** An anterolateral thigh (ALT) flap was raised at the left thigh, positioned on the soft tissue defect and the pedicle connected to the posterior tibial artery and veins by an end-to-end anastomosis using microsurgical techniques. **c** Postoperative findings after the successful ALT flap transfer

An anterolateral thigh (ALT) flap was raised at the left thigh with its perforator vessels together with a skin island according to the defect size and irrigated with diluted heparin solution until both veins returned clear fluid. The flap was then positioned on the soft tissue defect and the pedicle connected to the posterior tibial artery and veins by an end-to-end anastomosis using microsurgical techniques (Fig. 4a–c). In the same surgical session, the sustentaculum tali was reduced by a second team and fixed to the body of the calcaneus with two lag screws through the existing medial wound. Postoperatively, the extremity was kept warm and positioned in a hanging external fixator for 7 days.

The patient was kept non-weight bearing for 2 weeks followed by partial weight bearing with 20 kg until 12 weeks after surgery. The K-wires were removed after 6 weeks. Three months postoperatively a wound debridement was performed for a wound dehiscence at the distal end of the flap. The wound healed uneventfully in the following.

**Fig. 5:**
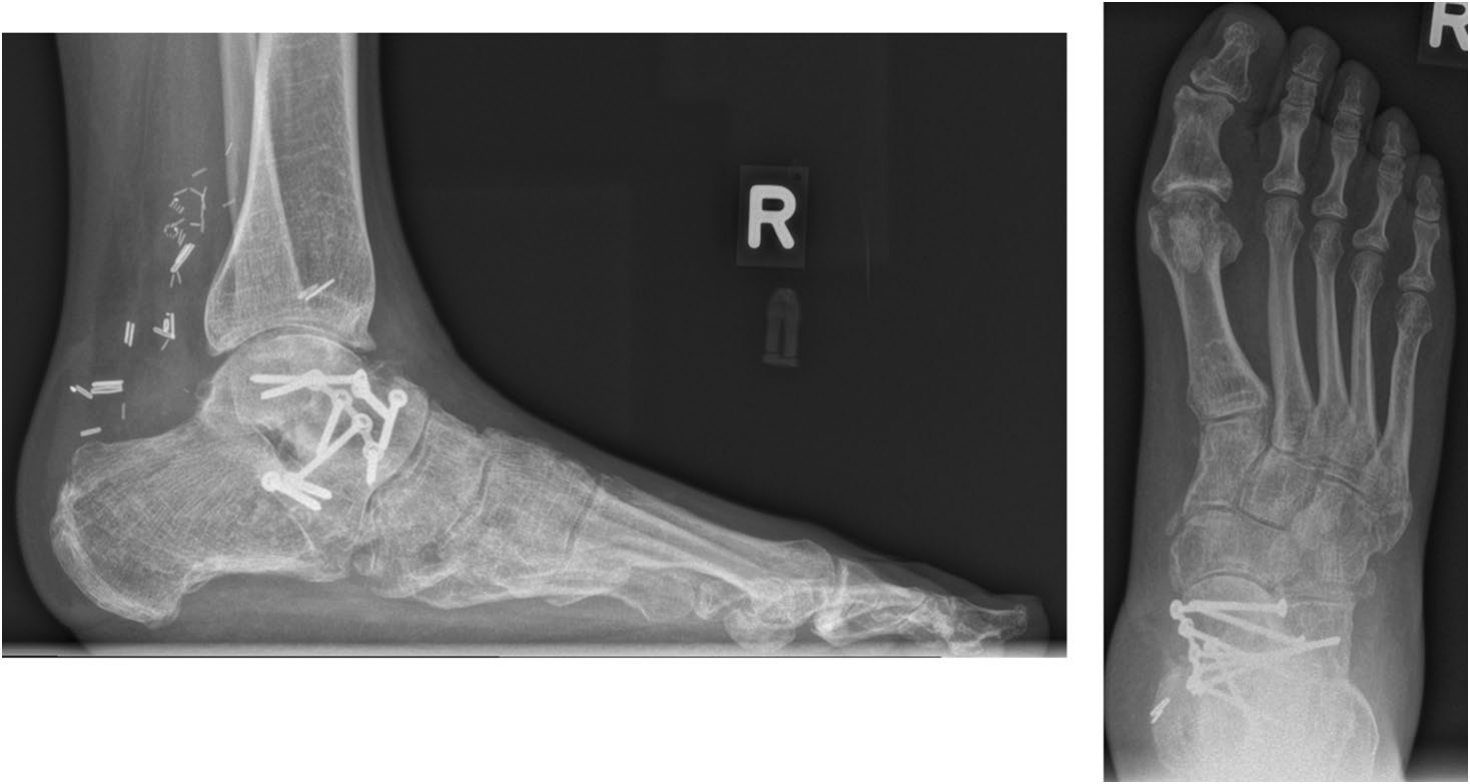
**a** Lateral view of the non-union of the talar neck postoperatively treated with debridement, cancellous bone grafting from the iliac crest, and medial plate fixation. **b** Dorsoplantar view postoperatively

Over the further course, a symptomatic non-union of the talar neck developed. The non-union was successfully treated with debridement, cancellous bone grafting from the iliac crest, and medial plate fixation 16 months following the injury (Fig. 5a, b). The patient returned to full weight-bearing after 12 weeks. Because of a conflict with shoewear, liposuction of the solidly healed flap that was performed at 20 months following the free ALT flap coverage.

**Fig. 6:**
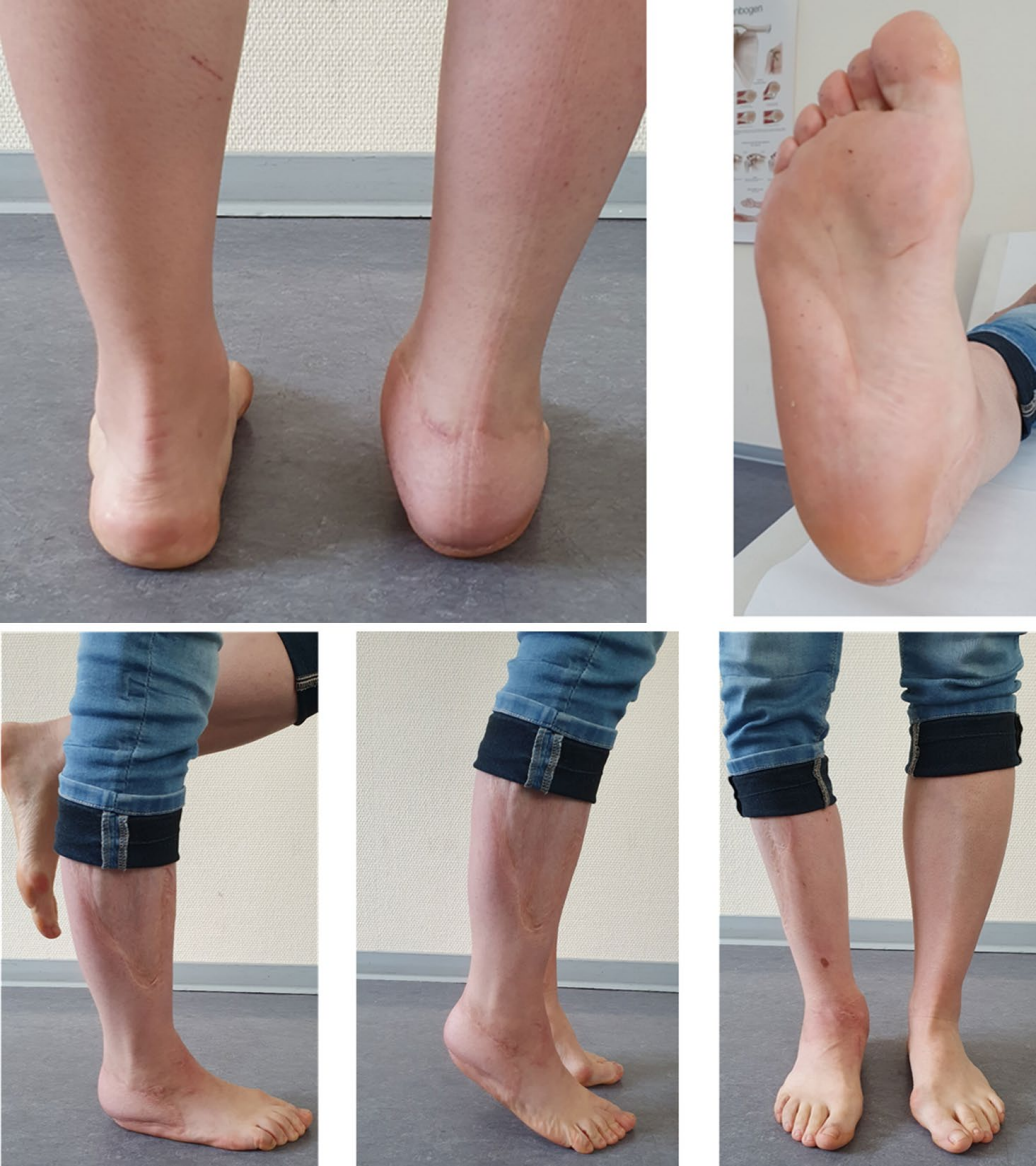
**a** Dorsal view of the heel pad, the patient was fully weight bearing in normal shoewear. **b** The soft tissues completely healed, the plantar heel pad was stable and solidly attached to the calcaneus. **c** The single leg stance is safe and not painful. **d** Dorsiflexion and plantarflexion of the foot were 10° and 20°. **e** Frontal view with the patient fully weight bearing

At final follow-up, 24 months after the injury, the patient was fully weight bearing in normal shoewear. She reported to be pain free during normal walking with a normal sensation over the plantar aspect of the heel. All wounds including the donor site at the thigh were completely healed. The plantar heel pad was stable and solidly attached to the calcaneus. Over the free flap and 1 cm below the former wound edges the patient could feel pressure on palpation. Dorsiflexion and plantarflexion of the foot were 10° and 20°, respectively. Eversion and inversion of the foot were restricted to 5° and 20°, respectively. The patient did not experience pain on active and passive motion. The single leg stance was safe and not painful (Fig. 6a–e).

## Discussion

Complete avulsion of the heel represents a severe soft tissue injury to the foot with a poor prognosis. In the past, even after successful initial operative treatment, it often resulted in a dysfunctional foot [1, 2, 21, 22]. In most cases of skin degloving injuries, the level of cleavage lies between the skin and the superficial fascia. At the heel, where there is a strong connection between the fascia and the periosteum, the disruption takes place between the fascia and bone. These wounds are characterized by large exposed areas and pronounced damage to the deeper tissue layers, such as tendons, nerves, blood vessels, bones, and joints. Despite surgical treatment, a non-vital and non-functional foot may persist, making amputation the only treatment option in many cases [6, 18].

Neither skin transfer, nor microsurgical coverage of the plantar heel with a free flap are sustainable concepts, as these structures do not have the physiological resistance and bony adherence of the original plantar tissue [17]. This leads to soft tissue damage and pain over the sole of the foot in the further course [6]. Therefore, due to the unique anatomy and function of the sole of the foot, every attempt should be made to preserve the original tissue.

We are aware of only few reports of successful reattachment of the sole of the foot. These procedures require an intact tibial neurovascular bundle, as well as the connection to remaining soft tissue bridges [1, 3, 12, 14, 15].

The basis for this technique is the blood supply to the sole of the foot described by Cichowitz et al. [23]. The vessels of the hindfoot form a subdermal and subperiosteal vascular plexus and run between the connective tissue septae connecting the plantar skin and the periosteum of the calcaneus. This anatomy allows simple refixation of the plantar fascia with K-wires if the soft tissue bridge is preserved [1]. Anchorage of the heel pad aims at creating strands of scar tissue resembling the septae of the heel pad while inducing minimal additional trauma to the avulsed heel pad. It, therefore, goes beyond simple suture with reattachment of the wound edges. The latter carries the risk of an unstable heel pad even if the tissue heals.

To the best of our knowledge, there is only one report on a similar technique by Cantrell et al. They described four cases of complete decollement of the sole of the foot treated with debridement, irrigation and degreasing of the leathered sole of the foot followed by fixation with Kirschner wires. We decided against degreasing of the heel pad. While it is known that degloving injuries can be successfully treated by degreasing the injured skin with subsequent reattachment [13, 17], defatting the skin of the sole will reduce the cushioning effect of the heel pad.

In the case presented here, we employed a double treatment strategy with anchorage of the completely avulsed heel pad with the use of multiple K-wires and microsurgical reconstruction of the full thickness soft tissue defect following skin necrosis at the posterior, non-weight-bearing aspect of the heel. The free flap healed uneventfully. A residual shoe conflict could be resolved with secondary debulking after the flap was judged to be durable.

Even though the soft tissue damage was the leading injury, the presented case was further complicated by displaced, intra-articular fractures of the talus and calcaneus. These are severe injuries even in isolation and are fraught with a guarded prognosis if they appear in combination [24, 25]. Nevertheless, even in these complex cases, favorable outcomes can be obtained with meticulous anatomic reconstruction of the alignment and joint surfaces at the hindfoot [26]. Complex foot trauma, defined as multiple level injury to the foot and ankle combined with severe soft tissue trauma requires a careful initial evaluation with respect to salvage vs. amputation and a multidisciplinary approach if the decision is made to salvage the extremity [26, 27]. In the case reported here, the joint effort of trauma, orthopedic and plastic surgeons resulted in a functional foot with a sensate, viable and durable soft tissue cover. We are not aware of a similar reported case in the literature.

In summary, this report demonstrates that anchorage with K-wires can be used as a simple and sufficient treatment option so salvage the heel pad and its unique properties even in case of complete avulsion. Skin breakdown and defects at the posterior aspect of the heel can be treated successfully with microsurgical free flap reconstruction because it is subject to less strain when walking. This combination of techniques together with primary suture of the ruptured anterior tibial tendon, anatomic reduction and internal fixation of the talar and calcaneal fractures lead to preservation of the soft tissue cover around the heel with good functional rehabilitation despite a complex injury.
